# Regional population and social welfare from the perspective of sustainability: Evaluation indicator, level measurement, and interaction mechanism

**DOI:** 10.1371/journal.pone.0296517

**Published:** 2024-01-11

**Authors:** Xueyi Wang, Mingchun Li, Taiyi He, Ke Li, Shengzhe Wang, Haoxiang Zhao

**Affiliations:** 1 Research Institute of Social Development, Southwestern University of Finance and Economics, Sichuan, China; 2 Law School, Southwest Petroleum University, Sichuan, China; 3 School of Land Resources and Environment, Jiangxi Agricultural University, Jiangxi, China; National Technical University of Athens: Ethniko Metsobio Polytechneio, GREECE

## Abstract

Key to regional sustainable development are the development and interplay of population dynamics and social welfare, each playing a significant role. As a representative region with demographic characteristics such as negative population growth and large labor outflow, the development and interaction between population and social welfare in Nanchong deserve in-depth exploration. This article takes the development of population and social welfare in Nanchong as the research object, and constructs an evaluation indicator system of population and social welfare through research backtracking, and uses entropy method and coupling coordination model to measure the development level and interactive effect of population and social welfare in Nanchong from 2010 to 2021. The research results show that: Firstly, the comprehensive evaluation results of population in Nanchong shows a linear upward trend, which indicates the stable positive effect of population structure and distribution, the gradual improvement effect of population quality effectively compensate for the weakening effect of population quantity, thus achieving the positive development of population. Secondly, the comprehensive evaluation results of social welfare in Nanchong shows an exponential upward trend, which indicates the social welfare has maintained a rapid growth momentum in various dimensions and the long-term positive effects have completely absorbed the negative effects, thus achieving the positive development of social welfare. Thirdly, during the sample period, the population and social welfare in Nanchong consistently maintained a high level of interaction strength, with factors diffusing and integrating. On this basis, the diffusion theory is used as an empirical reference to construct three interactive mechanisms between the population and social welfare in Nanchong and the implications are inferred from the empirical results.

## Introduction

As a general term for goals extracted from urgent practical needs, sustainable development shows the characteristics of planning and systematicity since proposed [1–3]. Over time, sustainable development has begun to transform into a novel concept that guides policies and related activities with its inherent advantages, and gradually gaining global attention [4–6]. Therefore, different regions with different characteristics have carried out a series of vivid practices closely related to sustainable development in a certain period [7]. Sustainable development has gradually evolved into a common pursuit of development, and the value of sustainable development has been widely recognized [8, 9]. Afterwards, in order to better promote the organic connection between sustainable development and regional demands, sustainable development began to shift from guiding objectives to a set of models, and continuously optimized its own structure in the practical process to better meet the numerous entities serving regions [10, 11].

When sustainable development is accepted and internalized as a code of action by many entities, and a model adapted to the positive evolution of the region is formed, the promotion of sustainable development faces new research points, that is, how to solidly promote sustainable development while considering the needs of the new era. By reviewing many studies, a common point that can be extracted is to systematically promote regional sustainable development by grasping and enhancing the sustainability of different elements within the region. Compared to sustainable development, sustainability focuses more on the ability to explain things, that is, to fill in more specific and rich information within the framework of sustainable development. Under the framework of the United Nations Sustainable Development Goals, globalization, technological innovation, trade openness, and renewable energy consumption have all played prominent roles in improving environmental quality and promoting ecological sustainability [12, 13]. Among them, green finance has a positive effect on sustainable economic growth [14], and opening up undertakings in the dimensions of economy, trade and culture, such as the One Belt and Road Initiative, also have a positive impact on the realization of sustainable economic, social and cultural goals in relevant regions [15]. At the level of technological innovation, technology is crucial in the development process [16]. With the development of higher education, the digital economy and artificial intelligence have shown unique advantages in reducing waste in economic and social activities [17, 18], improving energy efficiency and economic output efficiency, and are also important driving forces for promoting industrial development [19]. At the level of resource utilization, in the context of further promoting the construction of urban agglomerations and metropolitan areas, actively achieving efficient exchange and optimization of cross departmental energy consumption has provided more possibilities for exploring regional sustainable development paths [20]. At the level of environmental governance, there is significant spatial heterogeneity in the development of ecosystems, and human socio-economic activities also have a complex feedback effect on regional sustainability. Environmental governance can help improve regional ecological sustainability in terms of increasing vegetation coverage and reducing natural resource consumption [21]. At the level of climate change, several researches have formed a consensus that specific actions to address climate change, such as systematic planning and scientific deployment, are needed to enhance sustainability [22–25]. At the level of ecological status, the improvement of ecological governance capacity and the scientific setting of ecological development planning have significant contributions to regional sustainability [26–28].

Previous studies have shown that economic and financial changes, industrial development, technological innovation, resource utilization, environmental governance, climate change, and ecological status have significant contributions to the sustainability of regional internal factors. Among them, the sustainability of population and social welfare are seldom considered and discussed. As the foundation of regional sustainable development, population changes will fully shape the regional development pattern. Taking COVID-19 pandemic as an example [29], the burst of COVID-19 pandemic reduced the fertility willingness of the childbearing age group, increased the death risk of population, adjusted the education methods, suppressed population migration, thus having a comprehensive impact on population quantity, quality, structure, and distribution. So the sustainability of population needs to be taken seriously [30]. Measuring the complex system of population can effectively analyze the sustainable changes in population and clarify the progress mechanism within the framework of regional sustainable development [31]. As the security for regional sustainable development, changes in social welfare will effectively guide the allocation of regional resources, so the sustainability of social welfare needs to be taken seriously [32]. Measuring the diverse system of social welfare can effectively analyze the sustainable changes in social welfare and clarify the progress mechanism within the framework of regional sustainable development [33]. Population and social welfare are not isolated, but interconnected [34]. Therefore, there must be a certain interactive pattern between population and social welfare in the process of social development. The analysis of the Interaction effect and mechanism between population and social welfare will further provide explanations for enhancing the sustainability of regional development factors [35]. The existing sustainable development framework lacks explanatory power on the relationship between population and social welfare, and new theories are urgently needed to explain it. This article selects the diffusion theory of physics. The diffusion theory suggests that the continuous thermal movement of key substances between materials can cause mutual diffusion, leading to the formation of interwoven and firm bonds between materials. The strength of this bond increases to its maximum value over time [36]. Introducing diffusion theory into this study can provide evidence for the interaction between population and welfare systems and construct a theoretical framework with explanatory significance, integrating population and social welfare into the framework of regional sustainable development.

China has a huge national territory and significant regional differences. Therefore, selecting suitable regions as research samples in China’s development environment is very important. The reasons for choosing Nanchong in Sichuan Province for this study are as follows. Firstly, Nanchong is located in the western region, which is spatially underdeveloped. The discussion on sustainable development models in developed regions is more intense [37], and the guidance effect for underdeveloped regions is relatively lacking [38]. Therefore, the study of Nanchong can provide experience and reference for the sustainable development of underdeveloped areas. Secondly, the aging level in Nanchong remains relatively stable, with a large outflow of labor force, and a sustained negative population growth, which will have very typical significance from a demographic perspective [39]. Thirdly, the strategic arrangement of population and social welfare in Nanchong is relatively close, providing a suitable observation window [40].

Based on literature review, the question that this study aims to explore are as follows. Firstly, what is the level of population and social welfare development from the perspective of sustainability. Secondly, what is the interaction between population and social welfare under observable regional development conditions. Thirdly, what is the interaction mechanism between population and social welfare in representative regions.

The marginal contribution of this article is as follows. Firstly, scientifically Measuring the development level and interaction effect of population and social welfare in typical regions, and providing method reference and empirical numerical reference for the spatiotemporal evolution and interaction level of population and social welfare in the same type of research region. Secondly, using diffusion theory to explain the interaction mechanism between population and social welfare is conducive to fully clarifying the interactive conditions and trends of regional population and social welfare. On this basis, clarifying the explanatory space of diffusion theory, achieving a close correspondence between the theoretical and empirical development of typical regional population and social welfare from the perspective of sustainability.

## Method and data

**Fig 1:**
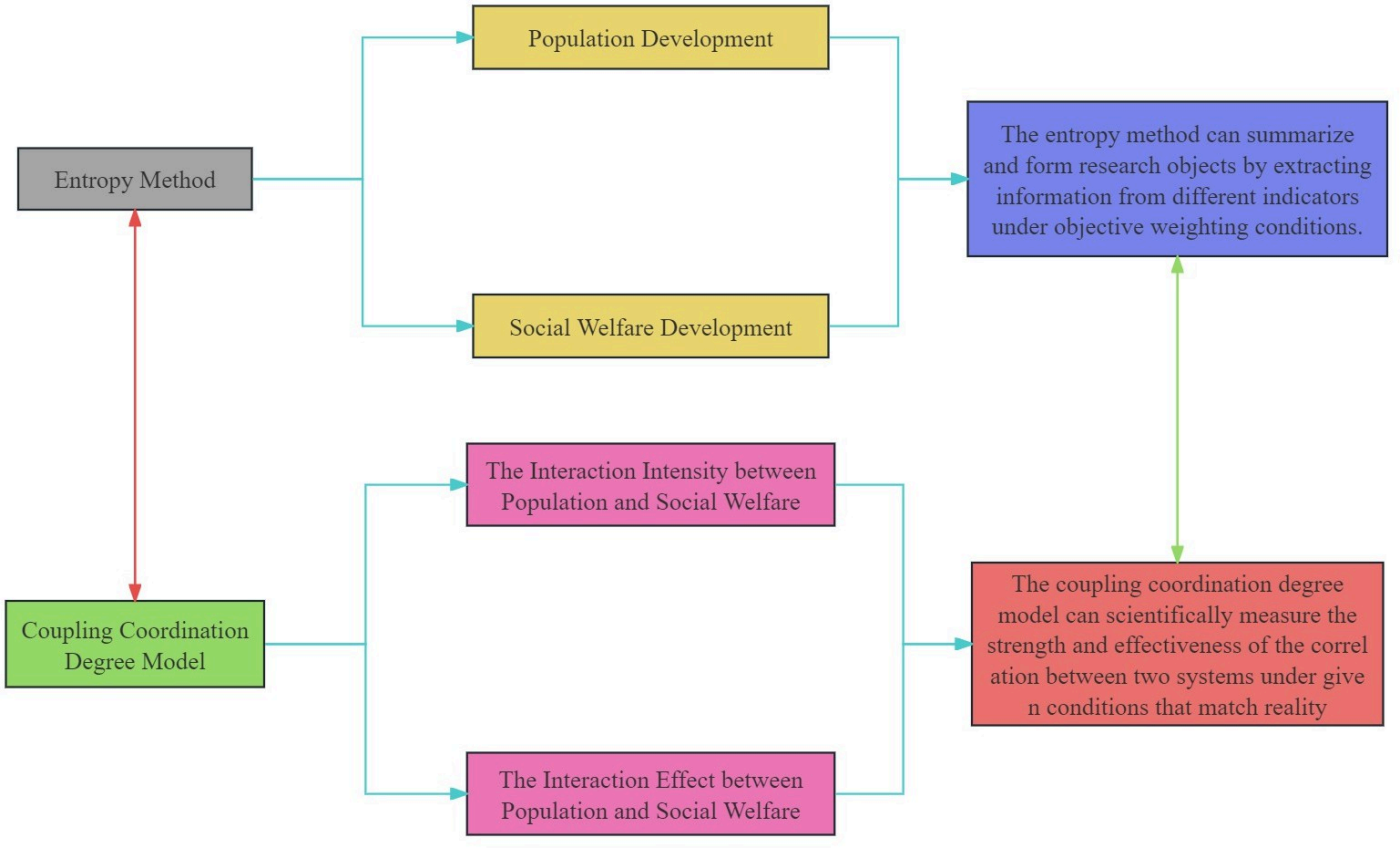
Method selection standard and applicability.

The selection standard and applicability of method in this study are shown in Fig 1. Afterwards, the principles of the research method will be explained.

### Entropy method

This study uses the entropy method to measure population and social welfare, which maximizes the scientific measurement of things under objective weighting conditions. The procedure are as follows.

Step1, normalize the initial data.

Separate treatment of positive and negative indicators:

$$R_{ij}=\frac{X_{ij}-\min(X_{j})}{\max(X_{j})-\min(X_{j})},\;0\leq R_{ij}\leq 1$$

$$R_{ij}=\frac{\max(X_{j})-X_{ij}}{\max(X_{j})-\min(X_{j})},\;0\leq R_{ij}\leq 1$$

R_ij_ is the standardized value of each indicator corresponding to each evaluation object, X_ij_(i = 1,2,⋯m; j = 1,2,⋯n) is the raw data for each indicator corresponding to each evaluation object.

Step 2, calculate the entropy of each indicator.

$$E_{j}=-\frac{1}{\ln(m)}\sum_{i}^{m}\frac{R_{ij}}{\sum_{i=1}^{m}R_{ij}}\cdot \ln\left(\frac{R_{ij}}{\sum_{i=1}^{m}R_{ij}}\right)$$

Step 3, the coefficient of variation D_j_ was calculated for each indicator.

$$D_{j}=1-E_{j}$$

Step 4, the weight W_j_ of each indicator is obtained.

$$W_{j}=\frac{D_{j}}{\sum D_{j}}$$

Step 5, the comprehensive score of single indicator.


$$S_{\mathrm{ij}}=W_{j}*R_{\mathrm{ij}}$$

### Coupling coordination degree model

According to the capacity coupling coefficient model, the following coupling function of the multi-system can be defined.

$$C_{n}={\left\{\left(u_{1}∙u_{2}∙\ldots ∙u_{n})/\Pi (u_{i}+u_{w}\right)\right\}}^{1/n},\;i\neq w,\;C\in [0,1]$$

$$u_{j}=\overset{n}{\underset{j=1}{\sum }}\lambda_{\mathrm{ij}}u_{\mathrm{ij}}\sum_{j=1}^{n}\lambda_{\mathrm{ij}}=1$$

u_i_(i = 1,2,…,m) is the evaluation index for the i subsystem, n is the number of systems involved in the synergy. u_ij_ is the j order parameter under the i subsystem and λ_ij_ is the weight of each order parameter.

Model the coupling degree of the population and social welfare.


$$C=2{\left\{(P∙W)/{\left(P+W\right)}^{2}\right\}}^{1/2}$$

Among them, C represents the coupling degree, P and W are the combined ordinal coefficients of the population system and the social welfare system, respectively. When all evaluation values are 0, the coupling degree is meaningless. When any evaluation value is 0, the coupling degree is 0.


$$\left\{ \begin{array}{c} D=(C∙T{)}^{1/n}\\ T=\sum_{i=1}^{n}\rho_{i}u_{i} \end{array}\right.$$

T is the comprehensive evaluation index of multiple systems, n is the number of subsystems, and ρ is a coefficient to be determined, depending on the importance of two systems to the overall coordinating role or the tendency of the research focus.

### Data source

The data source of this article is the Statistical Yearbook of Nanchong from 2011 to 2022.

### Evaluation indicator

#### Population

The definition of population is the collection of a certain number of people. This set is not only a simple addition in quantity, but also an ordered interweaving of multiple relationships. Therefore, population is a complex system, and any single indicator is difficult to fully reflect the sustainability of population development. Establishing a population evaluation indicator system can provide more objective and scientific calculations of population development. Based on a summary of previous researches, this study has decided to construct a population indicator evaluation system from four dimensions: population quantity, quality, structure, and distribution. The rationality of this construction lies in fully considering the dynamic changes in population development. The connotation of population quantity is the result of population changes over a certain period of time. In this study, the indicator of permanent population size is selected to represent population quantity, and its rationality lies in: on the one hand, the statistical subject of permanent population size is the National Bureau of Statistics, and the statistical source has strong authority and persuasiveness. On the other hand, the result of permanent population size is a direct reflection of the superposition of multiple effects such as birth, death, and mobility, which has strong practical representativeness. In addition, the permanent population size is closely related to the sustainability element of resource carrying capacity. The connotation of population quality is the result of changes in the comprehensive literacy of the population during the continuous evolution of civilization. This study selects the population in regular higher education institutions as an indicator to represent population quality. Its rationality lies in the fact that as an important place for human capital cultivation and transformation, the population in regular higher education institutions can effectively reflect the human capital stock that can be released in the region, thus explaining population quality at the level of realizing the value of human capital. In addition, the population in regular higher education institutions is closely related to the sustainability element of innovation. The connotation of population structure is the result of changes in the internal state of different types of population within a unit period. This study selects The ratio of employees in the secondary and tertiary industries to the total employees as an indicator to represent population structure. Its rationality lies in the fact that changes in the population industrial structure can fully reflect changes in the regional labor force structure in the context of aging and fewer children. At the same time, changes in the population industrial structure reflect changes in labor employment choices, which are closely related to the sustainability element of industrial transformation and upgrading. The connotation of population distribution is the result of population changes at the geographical and spatial level during the process of spatiotemporal evolution. This study selects the urbanization rate as an indicator to represent population distribution. Its rationality lies in that the urbanization rate is an intuitive result of the distribution of urban and rural populations, which can effectively reflect the flow preferences of population between urban and rural areas. In addition, the urbanization rate is closely related to the sustainability element of economic growth. The concrete evaluation indicators are shown in Table 1.

**Table 1:** Population indicator system.

Indicator	Second level indicator	Third level indicator	Indicator type	Indicator weight
Population	Population size	Permanent population size	Scale indicator	0.253
	Population quality	Population in regular higher education institutions	Scale indicator	0.229
	Population structure	The ratio of employees in the secondary and tertiary industries to the total employees	Structural indicator	0.178
	Population distribution	Urbanization rate	Structural indicator	0.340

#### Social welfare

The definition of social welfare is all measures taken to improve the material and cultural life of the general members of society, and it is a good state of life for the members of society. Therefore, social welfare is also a collective concept, which often does not have obvious exclusivity. The practical observation of social welfare must take the widespread participation and universal benefits of social members into consideration. A single indicator is difficult to fully reflect the sustainability of social welfare, so it is necessary to establish a social welfare evaluation indicator system. On the basis of summarizing previous research, this study has decided to construct a social welfare evaluation indicator system from eight levels: income level, consumption status, environmental input, social security, health resources, innovation support, employment status, and internet participation. The rationality of this construction lies in fully considering the inclusive effect of social welfare.The connotation of income level is the total value created by the population within a certain period of time. This study selects the indicator of per capita disposable income as a representative of income level, and its rationality lies in that per capita disposable income not only includes cash income but also physical income, which is a relatively complete observation of residents’ income level and an explicit social welfare. In addition, per capita disposable income is closely related to the sustainability element of economic prosperity. The connotation of consumption status is the numerical result of residents’ material needs over a certain period of time. This study selects the indicator of per capita consumption expenditure of urban residents to represent the consumption situation. Its rationality lies in that per capita consumption expenditure of urban residents intuitively displays the actual situation of the population at the consumption level, and is a characterization of the size of the regional consumption market and an explicit social welfare. In addition, per capita consumption expenditure of urban residents is closely related to the sustainability element of residents’ confidence in economic development. The connotation of environmental input is the collection of resources invested in promoting environmental development within a certain period of time. This study selects government expenditure on energy conservation and environmental protection as an indicator to represent environmental input. Its rationality lies in that as an important entity in budget formulation and fund allocation, government expenditure on energy conservation and environmental protection not only clarifies the boundaries of regional environmental development, but also influences the attitudes of other institutions towards environmental input by demonstrating the government’s attention to environmental protection, thereby shaping the overall trend of regional environmental development, It is a security element for the realization of social welfare. In addition, government expenditure on energy conservation and environmental protection is closely related to the sustainability element of green development. The connotation of social security is the result of changes in rights and interests during the continuous evolution of society. This study selects the indicator of the number of employees participating in pension insurance to represent social security. Its rationality lies in that the number of employees participating in pension insurance reflects the expansion of pension insurance coverage, and the comprehensive coverage of pension insurance will provide assistance for the improvement of the social security system under the trend of aging, which is a manifestation of the effect of social welfare. In addition, the number of employees participating in pension insurance is closely related to the sustainability element of group risk resistance. The connotation of health resources is the resources that the population can seek to achieve the accumulation of health capital and the improvement of health status within a certain period of time. This study selects the indicator of the number of beds in medical institutions per 10000 permanent residents to represent health resources. Its rationality lies in that the number of beds in medical institutions per 10000 permanent residents reflects the reserve capacity of medical resources to cope with the risk of population health status damage, plays a fundamental role in regional health resources, and is also an explicit social welfare. In addition, the number of beds in medical institutions per 10000 permanent residents is closely related to the sustainability element of shared development. The connotation of innovation support is the actions taken at the innovation level to achieve social welfare goals within a certain period of time. This study selects the number of researchers as an indicator to represent innovation support. Its rationality lies in the fact that the number of researchers is an innovative factor support to achieve social welfare for the population within the region, which can effectively weaken the barriers to social welfare through the diffusion effect of innovation, and is a promoting factor for welfare realization. In addition, the number of researchers is closely related to the sustainability element of innovative development. The connotation of employment status is the flow and allocation of labor market factors within a certain period of time. This study selects the unemployment rate as an indicator to represent the employment status. Its rationality lies in that the unemployment rate can intuitively reflect the problem of idle labor market resources, thereby indicating the dynamic changes in the number of people whose social welfare is weakened. It is a sensitive element that reflects the issue of social welfare realization. In addition, the unemployment rate is closely related to the sustainability element of coordinated development. The connotation of internet participation refers to the changes in population, internet access, experience, and other aspects during the process of social development. This study selects the number of international internet users as an indicator to represent internet participation. Its rationality lies in the fact that the number of international internet users is the population that can access broader information, and the essence of internet use by the population is to transform technology dividends into knowledge dividends, which is a result of pursuing social welfare. In addition, the number of international internet users is closely related to the sustainability element of open development. The concrete evaluation indicators are shown in Table 2.

**Table 2:** Social welfare indicator system.

Indicator	Second level indicator	Third level indicator	Indicator type	Indicator weight
	Income level	Per capita disposable income	Intensity indicator	0.103
	Consumption status	Per capita consumption expenditure of urban residents	Intensity indicator	0.107
	Environmental input	Government expenditure on energy conservation and environmental protection	Scale indicator	0.193
Social welfare	Social security	Number of employees participating in pension insurance	Scale indicator	0.144
	Health resource	Number of beds in medical institutions per 10000 permanent residents	Intensity indicator	0.113
	Innovation support	Number of Researchers	Scale indicator	0.069
	Employment status	Unemployment rate	Structural indicator	0.121
	Internet participation	Number of international internet users	Scale indicator	0.150

### Empirical results

#### Level measurement

**Fig 2:**
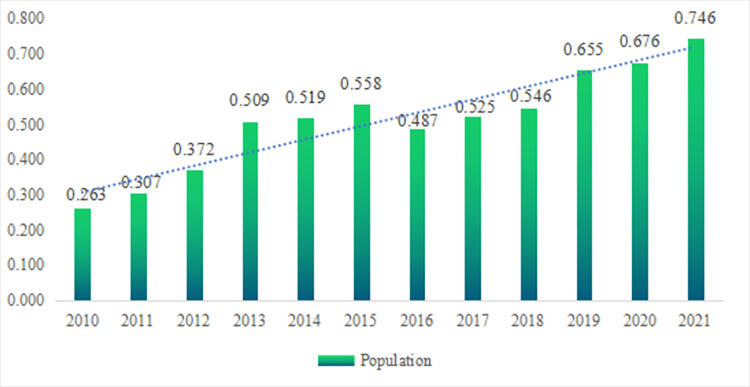
Population evaluation results in Nanchong.

As shown in Fig 2, the comprehensive evaluation results of population in Nanchong increased from 0.263 to 0.558 during the period from 2010 to 2015, indicating a rapid improvement in the level of population development. The strong momentum of population development during this period was attributed to the continuous increase in the ratio of employees in the secondary and tertiary industries to the total employees and the urbanization rate. At the same time, the negative population growth and the instability of population in regular higher education institutions had a restraining effect on the rapid growth of population development during this period. During the period from 2015 to 2016, the comprehensive evaluation results of population in Nanchong decreased from 0.558 to 0.487, indicating a slight decline in the level of population development. The decline in population development during this period was caused by the slowdown in the ratio of employees in the secondary and tertiary industries to the total employees and the urbanization rate, as well as the dual negative population growth and the population in regular higher education institutions. During the period from 2016 to 2021, the comprehensive evaluation results of population in Nanchong increased from 0.487 to 0.746, indicating a slow growth in the level of population development. The upward momentum of population development during this period was attributed to the steady increase in the population in regular higher education institutions, the ratio of employees in the secondary and tertiary industries to the total employees and the urbanization rate. At the same time, the inhibitory effect on the changes in the level of population development during this period was concentrated in the negative population growth. The experience that can be condensed from the above data results are as follows. Firstly, negative population growth is a prominent factor hindering the positive population development in Nanchong, but negative population growth cannot determine the trend of the comprehensive evaluation results of population. A rational view of negative population growth is a necessary measure to achieve regional sustainable development. Secondly, the negative changes in population quality have a time-varying effect on the hindering effect of population development in Nanchong, and maintaining an improvement in population quality remains the overall trend of development. At the same time, the positive changes in population structure and distribution have a steady promoting effect on the population development in Nanchong. Thirdly, as a typical region with obvious characteristics of labor outflow, the optimization of the industrial structure and adjustment of population spatial distribution in Nanchong can effectively compensate for the negative effects caused by the decrease in population quantity, unstable population quality, and human capital transformation. Fourthly, the comprehensive evaluation results of population are the result of the interaction and complex interweaving between the internal systems of the population. Reasonably grasping the regulation of population change and formulating matching population development strategies can greatly enhance the sustainability of population development.

**Fig 3:**
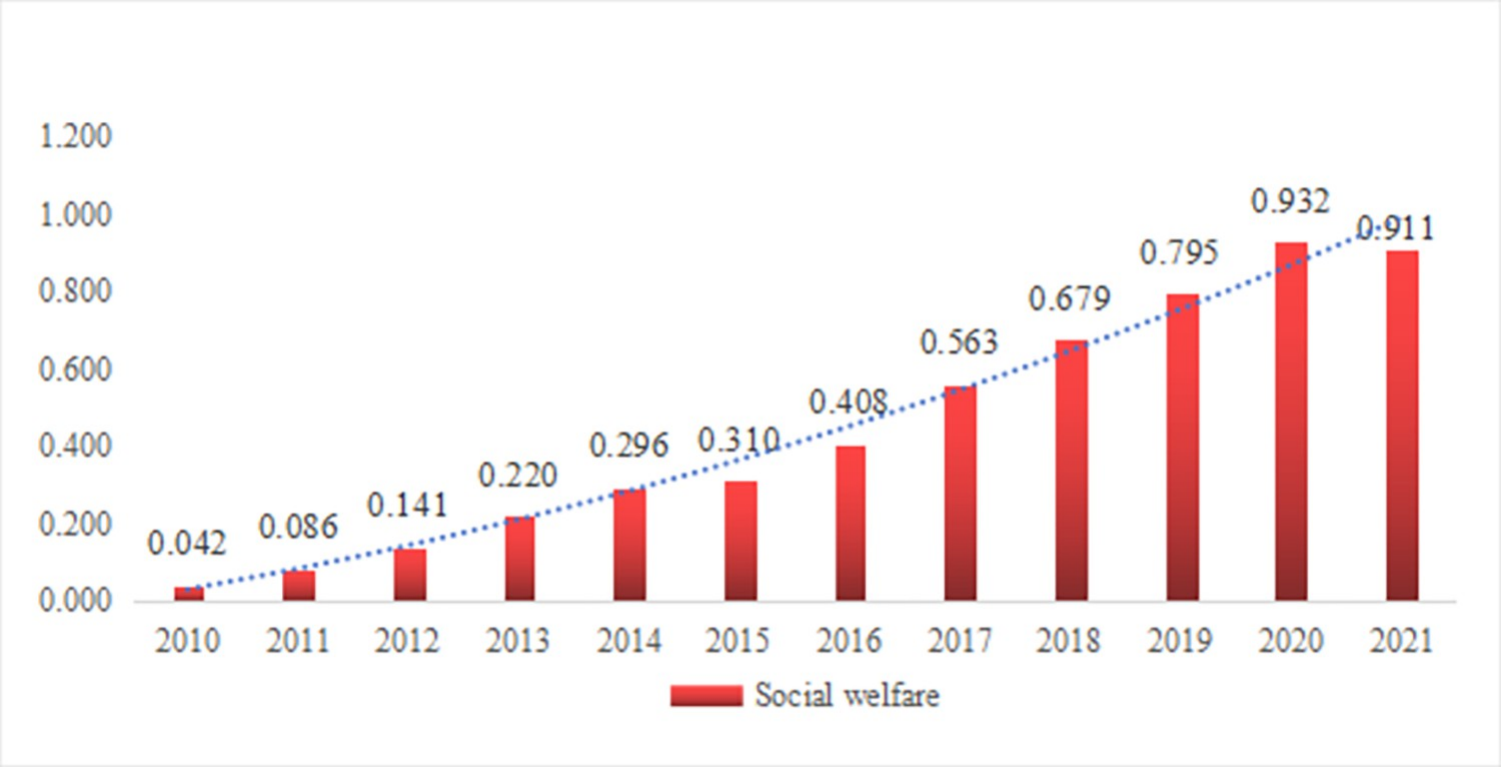
Social welfare evaluation results in Nanchong.

As shown in Fig 3, the comprehensive evaluation results of social welfare in Nanchong increased from 0.042 to 0.296 during the period from 2010 to 2014, indicating a gradual improvement in the level of social welfare development. The improvement of social welfare development during this period was attributed to the per capita disposable income, per capita consumption expenditure of urban residents, the number of beds in medical institutions per 10000 permanent residents, the number of researchers, the continuous increase in the number of international internet users, and the decrease in unemployment rate. At the same time, the fluctuations in government expenditure on energy conservation and environmental protection and the number of employees participating in pension insurance during this period have caused some interference with the gradual improvement of social welfare development. During the period from 2015 to 2021, the comprehensive evaluation results of social welfare in Nanchong increased from 0.310 to 0.911, indicating a rapid improvement in the level of social welfare development. The sharp increase in social welfare development during this period was attributed to per capita disposable income, per capita consumption expenditure of urban residents, the number of employees participating in pension insurance, the number of beds in medical institutions per 10000 permanent residents, and the steady increase shown by the number of international internet users; At the same time, government expenditure on energy conservation and environmental protection, the number of researchers, and changes in unemployment rate caused some interference with the sudden increase in the level of social welfare development during this period. The experience that can be condensed from the above data results are as follows. Firstly, the fluctuation of environmental input is a prominent factor hindering the positive social welfare development in Nanchong. However, environmental input itself is a moderate indicator and cannot determine the trend of the comprehensive evaluation results of social welfare. Dialectically treating the fluctuation of environmental input is an important path to achieve regional sustainable development. Secondly, the impact of changes in social security, innovation support, and employment status on the level of social welfare development in Nanchong has a time-varying effect. However, the expansion of social security coverage, the enhancement of innovation support, and the improvement of employment status are still the overall trend of development. At the same time, the positive changes in income level, consumption status, health resources, and internet participation have a steady promoting effect on the level of social welfare development in Nanchong. Thirdly, as a demonstration area with high attention to social welfare, the positive effects released by the changes in income level, consumption status, health resources, and internet participation in Nanchong can fully offset the unstable government input in environmental protection, partial decline in social security, and the negative effects brought by the intermittent disorder of innovation support and employment status. Thirdly, changes in the the comprehensive evaluation results of social welfare are the result of the interaction between internal systems of social welfare. Actively adapting to the changing regulation of social welfare and formulating forward-looking social welfare plans can greatly enhance the sustainability of social welfare development.

#### Coupling coordination

**Fig 4:**
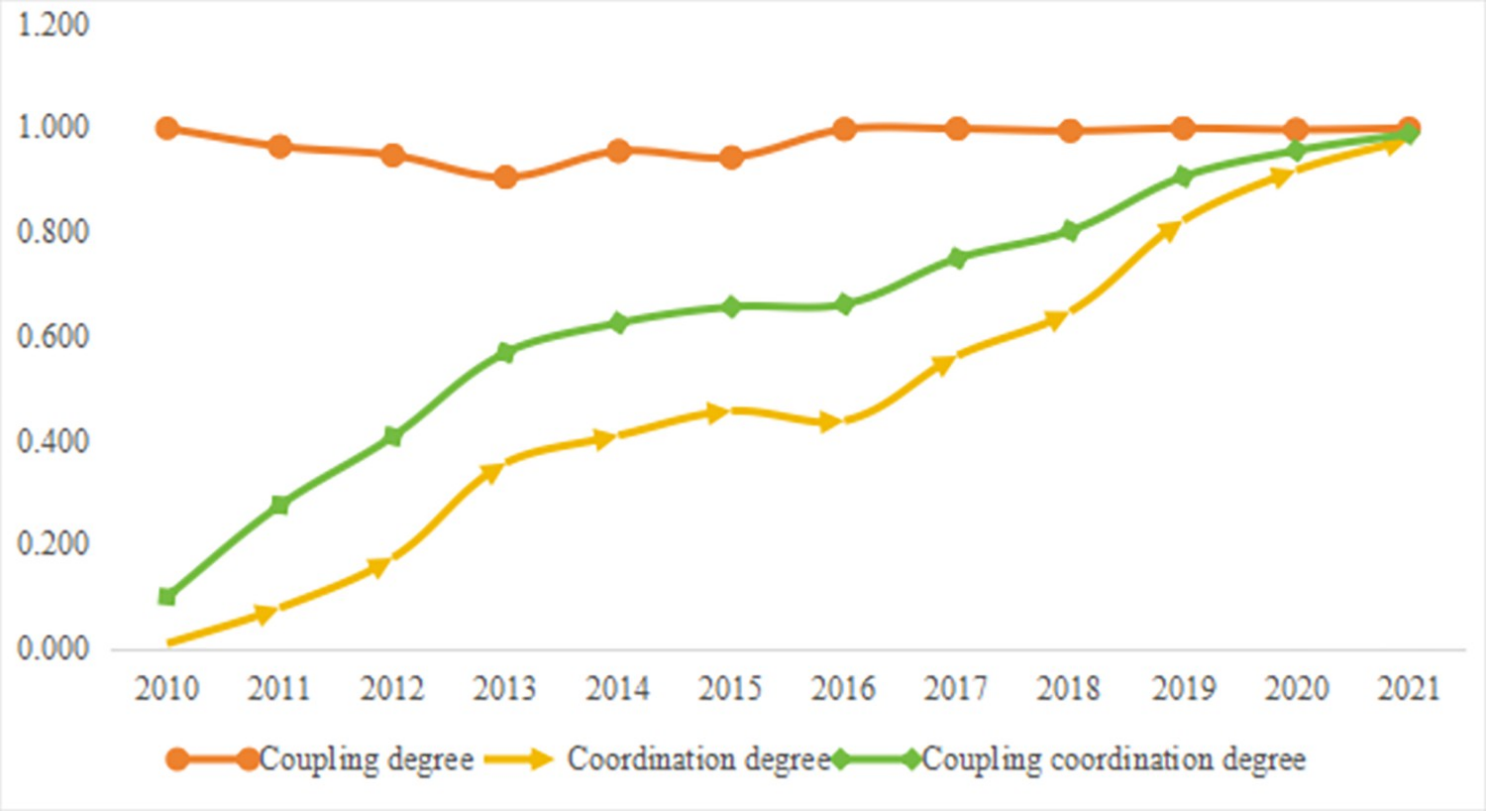
Coupling coordination results between population and social welfare in Nanchong.

As shown in Fig 4 and Table 3, firstly, the coupling degree between the population and social welfare in Nanchong remained at a high level from 2010 to 2021. This indicates that there is a strong connection between the population and social welfare in Nanchong, and there is a diffusion and integration of factors between the population and social welfare. Secondly, the coordination and coupling coordination between the population and social welfare in Nanchong from 2010 to 2021 showed a positive evolution trend. Specifically, the coordination degree increased from 0.010 (2010) to 0.979 (2021), with an overall increase of 9690%, while the coupling coordination degree increased from 0.100 (2010) to 0.989 (2021), with an overall increase of 889%. Further, the coordination proportion is significantly higher than the imbalance proportion in the results of coupling coordination degree, and the coupling coordination effect has undergone a transition from a severe disorder state to bare coordination to wonderful coordination. The above results fully indicate that the interactive effect between the population and social welfare in Nanchong exhibits an evolutionary feature of gradual optimization. Thirdly, the main driving force for the coupling coordination degree of population and social welfare in Nanchong has changed in different stages. During the period from 2010 to 2016, the results of the comprehensive evaluation of population were higher than those of the comprehensive evaluation of public social welfare. The improvement in the interaction effect between population and social welfare was attributed to the pulling effect of population on social welfare. During the period from 2017 to 2021, the results of the comprehensive evaluation of public social welfare were higher than those of the comprehensive evaluation of population. The improvement in the interaction effect between population and social welfare was attributed to the support effect of social welfare on the population.

**Table 3:** Coupling coordination results between population and social welfare in Nanchong.

Time	Coupling degree	Coordination degree	Coupling coordination degree	Effect
2010	1.000	0.010	0.100	Severe disorder
2011	0.965	0.079	0.276	Moderate disorder
2012	0.948	0.175	0.408	Border on disorder
2013	0.906	0.357	0.569	Bare coordination
2014	0.956	0.410	0.626	Primary coordination
2015	0.944	0.457	0.657	Primary coordination
2016	0.998	0.438	0.662	Primary coordination
2017	0.999	0.563	0.750	Intermediate coordination
2018	0.995	0.648	0.803	Good coordination
2019	1.000	0.823	0.907	Wonderful coordination
2020	0.997	0.919	0.957	Wonderful coordination
2021	1.000	0.979	0.989	Wonderful coordination

#### Interaction mechanism

**Fig 5:**
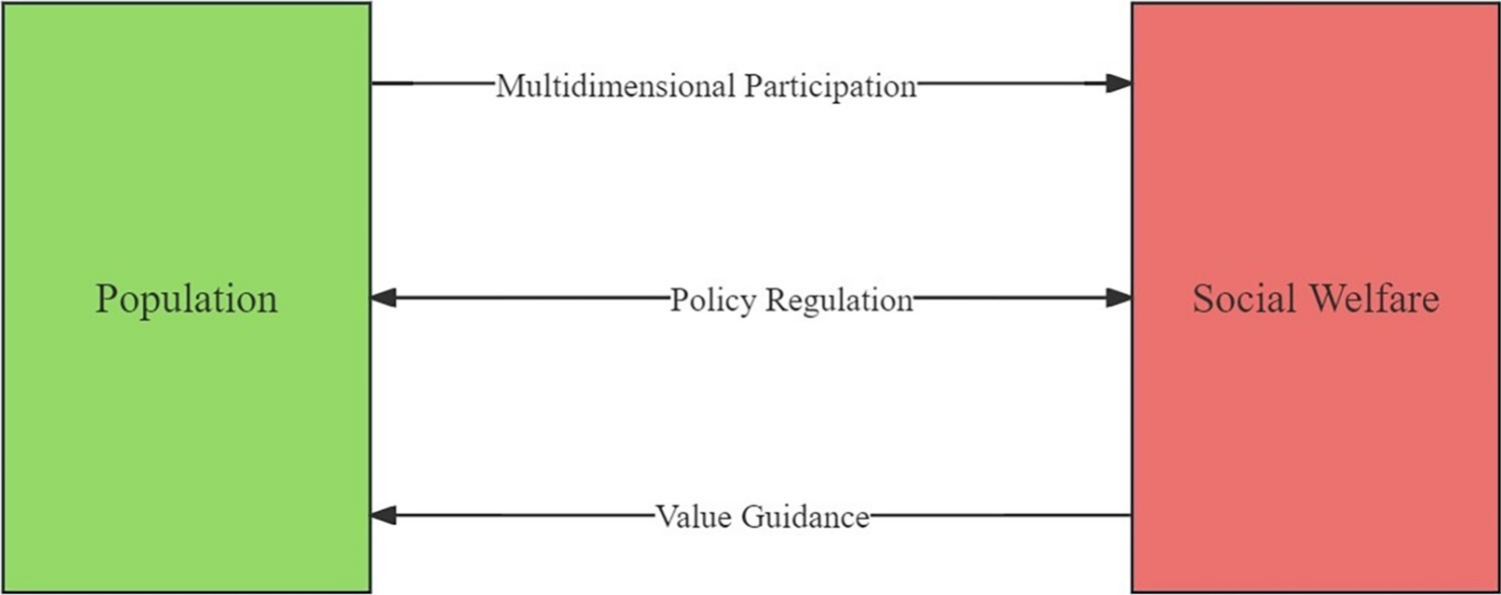
Interaction mechanism between population and social welfare in Nanchong.

As shown in Fig 5, there are three types of interaction mechanisms between population and social welfare.

The first is the multidimensional participation mechanism. The design starting point of this mechanism is the diffusion of population factors in social welfare. According to the diffusion theory based on which this study is based, there is a potential factor diffusion mechanism between population and social welfare. In the Nanchong, the release of human capital from labor will lead to an increase in per capita disposable income. The change in the willingness of the entire-age population to consume will affect the proportion of consumption expenditure. The change in population environmental attention will provide valuable reference for the adjustment of government expenditure on energy conservation and environmental protection. Negative population growth will force the improvement of the pension insurance system and alleviate the pressure on the allocation of medical resources to some extent. The improvement of population education level will provide a solid foundation for the increase of researcher scale. The optimization and upgrading of population industrial structure will effectively curb the rise of unemployment rate. The diversified model of population development will effectively prosper the emerging business forms represented by the Internet. In this mechanism, population is dominant, and the level of population development is often superior to the level of social welfare development.

The second is the policy regulation mechanism. The design starting point of this mechanism is the bidirectional factor diffusion between population and social welfare. According to the diffusion theory based on which this study is based, there may be a boundary between population and social welfare, leading to changes in the interaction effect between the two. In the Nanchong, macroeconomic policies integrate population and social welfare into the regional sustainable development framework by reducing information gaps, providing opportunities for bilateral information communication between population and social welfare, and balancing the bilateral interests of population and social welfare. In this mechanism, population and social welfare are compatible, and the level of population development and social welfare development is maintained in a balanced state.

The third is the value guidance mechanism. The design starting point of this mechanism is the diffusion of social welfare factors on the population. According to the diffusion theory based on which this study is based, there is a potential factor diffusion mechanism within social welfare towards the population. In the Nanchong, the increase in per capita disposable income will effectively boost the confidence of the entire-age population in economic development. The changes in consumption expenditure will reshape the population consumption pattern. The changes in government expenditure on energy conservation and environmental protection will have a targeted guiding effect on population environmental behavior. The expansion of pension insurance coverage will effectively mitigate pension risks. The increase in medical resource reserves will effectively meet the growing medical needs of the population. The increase in the researcher scale will provide a demonstration effect for the improvement of population quality. The changes in unemployment rate will affect the population’s career development choices and thus the employment structure. The entry of the Internet will effectively meet the demands of population development. In this mechanism, social welfare is dominant, and the level of social welfare development is often superior to the level of population development.

## Discussion

This study measures the development of population and social welfare in typical region and deeply analyzes the interaction mechanism between population and social welfare. The limitation of this research is that it focuses on the development and interaction framework between population and social welfare, without providing a detailed explanation of the antecedents and consequences of this framework. Therefore, based on research results and reflections, this article will further discuss from the following three aspects. Firstly, the factors influencing the development of the relationship between population and social welfare deserve in-depth consideration. The changes in population and social welfare are not only influenced by internal development, but also by external development [41, 42]. How to accurately identify and scientifically measure the impact of external factors will be a topic worth exploring. Secondly, it is worth exploring the coordinated promotion of population and social welfare development. From existing experience [43–46], there is always a mismatch between population and social welfare as two parts of interest linkage. Therefore, a mechanism is needed to coordinate the development differences between the two and reduce the problems caused by imbalanced development. How to design a collaborative mechanism is worth analyzing in the following research. Thirdly, the practical effects of population and social welfare development urgently need to be discussed. Looking back at past research, the relevant results can provide development references for third-party plans to a certain extent, and also lead to research topics worth further attention [47, 48]. In this study, the sustainable driving force support mechanism and effect evaluation of the positive changes in the population internal system on regional economic development, the impact mechanism and effect evaluation of the completeness of social welfare policies and the implementation effect of measures on the happiness and sense of achievement of different groups, and other issues can be expected to emerge under the condition of available data.

## Conclusions and implications

### Conclusions

Firstly, the comprehensive evaluation results of population in Nanchong have increased from 0.263 to 0.746 during 2010 and 2021, showing a linear upward trend in population development. The positive population development in Nanchong benefits from the optimization of population industrial structure and the adjustment of population spatial distribution, and constrains by negative population growth. Changes in population quality plays an important regulatory role in it. The key to maintaining the sustainability of population development lies in keeping the population structure, spatial distribution, and regional development needs in line, while the difficulty lies in how to compensate for the impact of negative population growth by improving population quality and human capital conversion efficiency.

Secondly, the comprehensive evaluation results of social welfare in Nanchong have increased from 0.042 to 0.911 during 2010 and 2021, showing an exponential upward trend in social welfare development. The positive social welfare development in Nanchong benefits from the positive changes in residents’ income level, consumption status, health resources, and internet participation, constrains by fluctuations in environmental input. Changes in social security, innovation support, and employment status play an important regulatory role in this. The key to maintaining the sustainability of social welfare is to enhance the sense of access to social welfare for the population, while the difficulty lies in improving the environmental status and security mechanisms for population welfare access.

Thirdly, during the sample period, the population and social welfare in Nanchong consistently maintained a high level of interaction strength, with factors diffusing and integrating. At the same time, the interactive effect between the population and social welfare in Nanchong shows an evolutionary feature of gradual optimization. The key to maintaining the coordinated development and wonderful coupling of population and social welfare lies in grasping the cyclical patterns of the interaction between population and social welfare and utilizing policy tools to achieve advantage superposition.

### Implications

Firstly, reasonably grasping the regulation of population changes, strengthen rational understanding of negative population growth and labor outflow, and formulate a matching population development strategy in Nanchong. It is encouraged to actively optimize the industrial structure, guide the population to gather in areas with high industrial demand, in order to compensate for the attack of negative population growth. At the same time, it is necessary to formulate scientific regional development policies, pay attention to the balance of development between different regions, and focus on the allocation of education, medical resources, transportation communication development, cultural and leisure facilities construction, and real estate policies, which can reasonably adjust the population spatial distribution in Nanchong. Further, continuously improving the population quality of Nanchong by providing more vocational training and skill learning opportunities and optimizing the population structure through flexible migrated population management in Nanchong.

Secondly, attaching importance to the interaction between the internal systems of social welfare in Nanchong, adapting to their changing patterns, and achieving the sustainability of social welfare. On the one hand, it is necessary to establish a sound environmental monitoring system, increase investment in clean energy, promote the development and utilization of renewable energy, encourage innovation and application of environmental protection technologies, promote the development of sustainable buildings and transportation systems, and reduce energy consumption and environmental pollution. In addition, it is necessary to actively participate in international environmental cooperation, learn and introduce advanced environmental technology and management experience, jointly address cross-border pollution and climate change, and promote international environmental cooperation. On the other hand, it is necessary to consider establishing a flexible social security system to adapt to changes in different industries, work forms, and employment relationships, and regularly adjust social security funds to adapt to changes in inflation and living costs. It is also required to strengthen innovation support, reduce the burden on small and micro enterprises, provide them with more development opportunities, and promote more new forms of employment, establish social consensus and cooperation, in order to optimize the employment environment and improve the level of social welfare development.

Thirdly, focusing on the interactive mechanism between the population and social welfare in Nanchong, promote the positive interaction between population and social welfare, strengthen the pulling effect of population on social welfare and the support effect of social welfare on population to promote sustainable urban development. It is not only necessary to implement flexible registered residence policies, encourage talents to settle in Nanchong, promote urban industrial upgrading, cultivate new economic growth points and attract investment to achieve the goal of promoting employment and population return, but also to increase social welfare investment, strengthen the retention policy for migrant labor, improve residents’ living standards and provide better public services to retain local residents.

## Supporting information

S1 Data (XLSX)
